# Evaluation of Postoperative Kyphotic Changes in Patients Who Underwent Cervical Laminectomy

**DOI:** 10.7759/cureus.73034

**Published:** 2024-11-05

**Authors:** Ahmed Abduljabbar Omar

**Affiliations:** 1 Department of Surgery, College of Medicine, Hawler Medical University, Erbil, IRQ

**Keywords:** cervical canal stenosis, kyphosis progression, laminectomy, neurological outcomes, spondylotic myelopathy

## Abstract

Background: The stenosis of the cervical canal due to spondylotic changes is one of the common causes of spinal cord compression. Without adequate treatment, it results in progressive neurological deterioration. However, despite the wide acceptance of newer techniques, such as laminoplasty and laminectomy with fusion, in most situations, especially in resource-constraint situations, the role of laminectomy alone is pertinent. The following study reviews the effectiveness and safety of laminectomy alone in patients with cervical spondylotic myelopathy.

Methods: A retrospective cohort study was conducted at Par Private Hospital, Erbil, including 46 patients diagnosed with cervical canal stenosis. All patients underwent laminectomy without fusion or laminoplasty, and postoperative outcomes were assessed using the modified Japanese Orthopaedic Association (mJOA) score, Neck Disability Index (NDI), and radiographic evaluations of cervical alignment. The relationship between preoperative and postoperative cervical alignment, kyphosis progression, and clinical outcomes was analyzed.

Results: Most patients showed significant neurological improvement in accordance with the improvement of scores of both mJOA and NDI. Among them, radiographic kyphosis progression occurred in 40% of patients, which was not associated with clinical deterioration. This showed a remarkable correlation between preoperative cervical alignment and postoperative outcomes, with R² = 0.733 and p < 0.001. No major complications like C5 palsy or wound infection were recorded throughout the follow-up period.

Conclusion: Laminectomy alone constitutes a proper and safe surgical alternative for cervical canal stenosis, especially in resource-poor countries. Although kyphosis progression occurred in some cases, there was no clinical impairment. Careful selection of patients by preoperative alignment is important for favorable outcomes. Comparing laminectomy alone with other techniques in longer follow-up will provide the final result in larger, multi-institutional groups.

## Introduction

Cervical spondylotic myelopathy (CSM) is one of the most common causes of spinal cord dysfunction in adults, often resulting from degenerative changes in the cervical spine that lead to spinal canal stenosis and subsequent compression of the spinal cord [1]. The clinical presentation of CSM can vary, ranging from mild sensory disturbances to severe motor dysfunction, making timely surgical intervention critical to prevent further neurological decline [2].

A number of surgical methods have been developed to decompress the spinal cord and arrest the progression of the disease. Laminectomy, a surgical procedure that entails partial removal of the vertebra to enlarge the spinal canal, has conventionally been the surgery for CSM. However, there are concerns about postlaminectomy kyphosis and segmental instability associated with the procedure, which often warrant the use of adjunct fusion techniques to minimize the risks of such complications. Fusion has the advantage of providing stability to the spine but at the cost of losing mobility as well as some possible complications such as hardware failure and additional segment degeneration [1].

Laminoplasty serves to decompress the spinal cord with the preservation of the posterior elements and spinal stability. This is particularly believed to be useful in cases where there is a great concern about spinal instability. It has been shown that this can have fewer postoperative deformities compared to laminectomy and, therefore, this is preferred in patients who are at risk for instability [3]. While both techniques have demonstrated efficacy in relieving symptoms of CSM, they differ significantly in terms of surgical outcomes and complications. Meta-analyses have shown that while both laminectomy and laminoplasty can result in clinical improvement, laminoplasty may result in fewer complications, such as C5 radiculopathy and superficial infection [1]. Besides this, several cost analyses have proved that laminoplasty is significantly less expensive than laminectomy with fusion; thus, it is quite feasible for resource-poor health systems [2]. On the other hand, laminectomy with fusion, while more invasive, demonstrates better outcomes in pain relief and overall functional improvement specific to cervical spine-related symptoms. However, this approach also carries a higher complication rate, as noted in studies utilizing the Core Outcome Measures Index for cervical spine conditions [4].

In light of the recent debate about the best surgical approach to CSM, laminectomy alone - without fusion or laminoplasty - is here presented in the treatment of cervical canal stenosis due to spondylotic changes. The investigation was performed in Iraq and thereby presents an opportunity to observe this treatment outcome in a developing healthcare system, in which treatment options may be heavily based on cost and other related factors concerning surgical experience.

## Materials and methods

Study design and patient population

This retrospective, observational cohort study was conducted at Par Private Hospital, Iraq. Data collection was done between 1 April 2024 and 1 August 2024. Patients who had undergone laminectomy for cervical canal stenosis due to spondylotic changes were included, with surgeries performed between 2021 and January 2024. In the clinical course, patients were selected with symptoms of CSM confirmed by MRI imaging. The necessary inclusion of patients was that they presented with progressive neurological deficits that required surgical intervention.

Patients who had previous surgery of the cervical spine, trauma, infection, or tumors were excluded from this study. The study was conducted within the institutional environment. Being a single-center study, all surgical procedures were done by the same team of neurosurgeons, thus minimizing variations in the rationale of surgical technique and postoperative care. Although this may ensure consistency, the single-center nature of this study may not allow the generalization of results to other healthcare settings.

Because this was a retrospective cohort study, the sample size depended on eligible patients according to the inclusion and exclusion criteria during the period of the study. No formal power analysis was performed; however, a sample size of 46 patients was adequate to produce information with meaningful postoperative outcomes, kyphosis progression, and complication rates. Larger samples may be required in subsequent studies to increase statistical power and generalizability.

Surgical procedure

All patients underwent a posterior cervical laminectomy alone, without the addition of fusion or laminoplasty. The decision to perform laminectomy in isolation was made by the attending neurosurgeon based on preoperative imaging studies, clinical presentation, and lack of evidence demonstrating spinal instability. Patients were then positioned prone under general anesthesia and a midline posterior cervical incision was made.

Paraspinal muscles were retracted to expose the lamina, which was removed using a high-speed burr at the affected cervical levels (C2-C7), ensuring decompression of the spinal canal while preserving the facet joints. No fusion hardware was placed, and the dura was inspected for adequate decompression before wound closure in layers. Postoperative radiographs were obtained immediately to confirm spinal alignment and monitor for any instability or kyphosis progression.

Follow-up protocol

The postoperative follow-up care included regular clinical and radiographic assessments. Patients were followed up after six weeks, three months, and six months, with yearly follow-ups depending upon their clinical condition thereafter. Neurological examination during follow-up included assessment of motor power, sensation, and reflexes at every visit.

Functional outcomes were measured using the modified Japanese Orthopaedic Association (mJOA) score [5], and the Neck Disability Index (NDI) [6] was used to assess neck-related disability. Radiographs were obtained postoperatively and at six-month intervals to monitor cervical lordosis and detect any kyphosis progression, using the Cobb angle measurement between C2 and C7 on lateral X-rays.

Complication monitoring and management

The patients were followed up carefully during their stay in the hospital and at follow-up for postoperative complications. These complications included C5 palsy, defined as postoperative weakness of the deltoid or biceps muscles, monitored clinically and treated conservatively with physical therapy; further imaging or surgical intervention was considered if there was persistent or worsening weakness. Wound infection, ranging from superficial to deep, was diagnosed in the presence of clinical signs such as erythema, warmth, or discharge.

All suspected infections were treated with antibiotics. Suspected cases of more serious infection were treated with surgical debridement. Kyphosis progression, representing the main complication of interest in this study, was defined as a loss of cervical lordosis or a transition into kyphosis as evidenced by follow-up radiographs. This was followed radiographically, and conservatively or surgically treated based on symptom severity in patients clinically presenting neck pain or neurological deficits due to kyphosis progression. The other complications were general neurological deteriorations, for which immediate assessment and imaging to guide management were obtained.

Radiographic and clinical outcome measures

The primary outcome measure was the improvement in the mJOA score, which evaluates neurological function, including motor and sensory domains. Secondary outcomes included changes in the NDI score, reflecting the patient’s neck-specific disability, and radiographic changes in cervical alignment (measured by the C2-C7 Cobb angle). Radiographic kyphosis progression was specifically tracked over time. Clinical improvements and the occurrence of postoperative complications, including C5 palsy and infection, were also documented.

Statistical analysis

Descriptive statistics were calculated for demographic and clinical variables, including age, sex, number of levels decompressed, and length of follow-up. The frequency of complications and kyphosis progression was expressed as a percentage for categorical variables. Means with standard deviations were used to describe mJOA scores and radiographic measurements for continuous variables.

Chi-square tests were used to determine the significance of relationships between categorical variables, including those between the presence of kyphosis progression and the number of levels decompressed. Linear regression analysis was performed to assess the relationship between preoperative and postoperative C2-C7 angulation.

Additionally, the number of levels decompressed and follow-up duration were included as covariates to determine their effects on postoperative outcomes. Multinomial logistic regression was used to explore how age and the number of decompressed levels impacted clinical outcomes, such as neurological recovery and kyphosis progression. A p-value of less than 0.05 was considered statistically significant. All statistical analyses were conducted using Jamovi version 2.3 and R version 4.1 (R Foundation for Statistical Computing, Vienna, Austria) [7,8].

Ethical considerations

This study was approved by the Institutional Review Board of Hawler Medical University on the 26th of March 2024 (Approval No.: 10). Informed consent was obtained from all patients included in the study. All procedures were in complete view and performed in accordance with standards for a human subject as stated by the Declaration of Helsinki in 1964 and its later amendments.

## Results

Patient demographics

**Figure 1:**
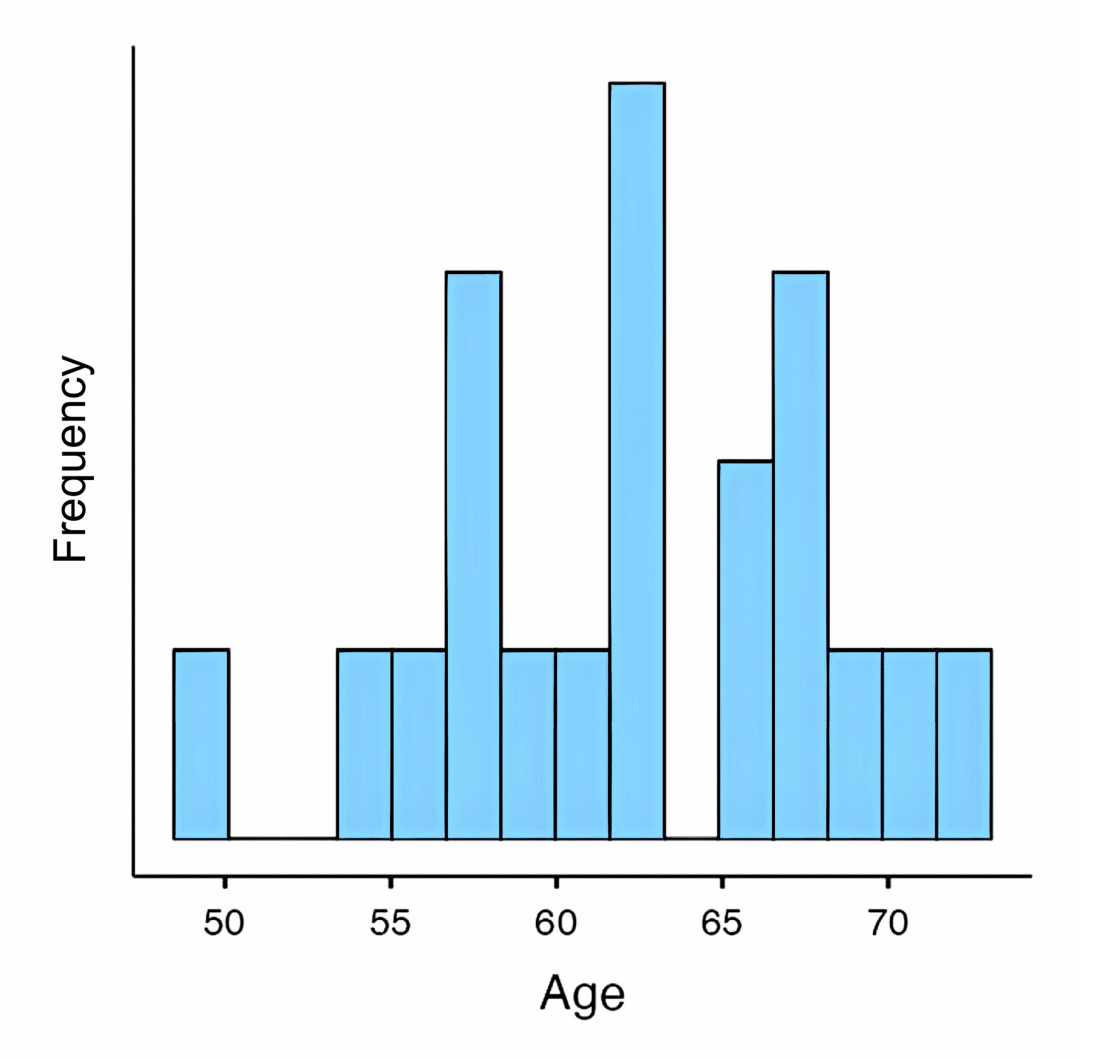
Age distribution of patients undergoing laminectomy for cervical stenosis.

A total of 46 patients underwent surgical treatment for cervical canal stenosis at Par Private Hospital. The average age of the patients was 62.3 years (SD = 5.78, range = 50-73) (Figure 1). The patient population consisted mostly of older adults presenting with degenerative cervical spinal stenosis. The majority of patients (93.5%, n = 43) were diagnosed solely with cervical stenosis, while a smaller proportion presented with additional pathologies such as combined cervical and lumbar stenosis (2.2%, n = 1) or cervical disc herniation (2.2%, n = 1).

One patient (2.2%) was diagnosed with cervical stenosis with ossification of the posterior longitudinal ligament (OPLL) (Table 1). These demographics are reflective of the typical presentation of CSM in older adults, where cervical stenosis due to degenerative changes is the predominant pathology.

**Table 1:** Frequencies of diagnosis. OPLL: ossification of the posterior longitudinal ligament.

Diagnosis	Counts	% of total	Cumulative %
Cervical and lumbar stenosis	1	2.2%	2.2%
Cervical disc	1	2.2%	4.3%
Cervical stenosis	43	93.5%	97.8%
Cervical stenosis OPLL	1	2.2%	100.0%

Surgical procedures

The surgical strategy in all cases consisted of a laminectomy with no adjunct fusion or laminoplasty (Tables 2, 3). Various laminectomy levels were performed depending on the extension of cervical stenosis. The most frequent stages of surgery included laminectomy from above C3/4 to below C6/7 (8.7%, n = 4).

**Table 2:** Frequencies of laminectomy and related surgical procedures in cervical stenosis patients.

Surgery type	Count	% of total
C3/4 laminectomy	1	2.2%
C3/4/5 laminectomy (partial C2 reached above C2/3 space)	1	2.2%
C3/4/5/6 and upper part of C7 laminectomy	1	2.2%
C3/4/5/6 laminectomy	3	6.5%
C3/4/5/6/7 laminectomy	1	2.2%
C4/5/6 laminectomy	3	6.5%
C5/6 laminectomy	1	2.2%
C6/7 discectomy with fusion spinart	1	2.2%
Laminectomy from C2/3 to below C6/7	1	2.2%
Laminectomy from above C2/3 to below C6/7	3	6.5%
Laminectomy from above C3/4 to below C6/7	4	8.7%
Laminectomy from below C2/3 to below C6/7	1	2.2%
Laminectomy from above C3/4 to below C5/6	1	2.2%
Laminectomy of C5/6 with partial C4 and C7 (L1/2 fixation and discectomy)	1	2.2%
Other laminectomy procedures (various levels)	20	43.5%

**Table 3:** Contingency table chi-square test between the diagnosed cases and surgeries.

Surgery	Counts	% of total	Cumulative %
C3/4 laminectomy	1	2.2%	2.2%
C3/4/5 laminectomy, partial C2, reached above C2/3 space	1	2.2%	4.3%
C3/4/5/6 laminectomy	3	6.5%	10.9%
C3/4/5/6/7 laminectomy	3	6.5%	17.4%
C3/5/6 laminectomy	1	2.2%	19.6%
C4/5/6 laminectomy	3	6.5%	26.1%
C5/6 laminectomy	1	2.2%	28.3%
C5/6 laminectomy, partial C4 and C7	1	2.2%	30.4%
C6/7 discectomy with fusion spinart	1	2.2%	32.6%
Cervical laminectomy	1	2.2%	34.8%
laminectomy from C2/3 to C4/5	2	4.3%	39.1%
laminectomy from C2/3 to below C6/7	1	2.2%	41.3%
laminectomy from C2/3 to below C7	1	2.2%	43.5%
laminectomy from C3/4 to C5/6	1	2.2%	45.7%
laminectomy from C3/4 to below C5/6	1	2.2%	47.8%
laminectomy from C3/4 to below C6/7 levels	1	2.2%	50.0%
laminectomy from C4/5 to C6/7	1	2.2%	52.2%
laminectomy from C4/5 to below C6/7	1	2.2%	54.3%
laminectomy from above C2 to below C4/5	1	2.2%	56.5%
laminectomy from above C2/3 to below C6/7	3	6.5%	63.0%
laminectomy from above C2/3 to below it	1	2.2%	65.2%
laminectomy from above C3/4 to C6/7	1	2.2%	67.4%
laminectomy from above C3/4 to below C4/5	1	2.2%	69.6%
laminectomy from above C3/4 to below C5/6	1	2.2%	71.7%
laminectomy from above C3/4 to below C6/7	4	8.7%	80.4%
laminectomy from above C3/4 to below C6/7 with hemilaminectomy of C5	1	2.2%	82.6%
laminectomy from above C3/4 to below it and from above C5/6 to below it	1	2.2%	84.8%
laminectomy from above C4/5 to below C5/6	1	2.2%	87.0%
laminectomy from above C4/5 to below C6/7	3	6.5%	93.5%
laminectomy from below C2/3 to below C6/7	1	2.2%	95.7%
laminectomy of C2/3/4/5 and upper lip of C6	1	2.2%	97.8%
laminectomy of C5/C6 with partial C4 and C7, L1/2 fixation and left discectomy, L4/5 left over the top decompression	1	2.2%	100.0%

Other common procedures included C3/4/5/6 laminectomy (6.5%, n = 3), C4/5/6 laminectomy (6.5%, n = 3), and laminectomy from above C2/3 to below C6/7 (6.5%, n = 3). Additionally, C3/4 laminectomy (2.2%, n = 1), C3/4/5 laminectomy partial C2 reached above C2/3 space (2.2%, n = 1), C5/6 laminectomy (2.2%, n = 1), C6/7 discectomy with fusion spinart (2.2%, n = 1), and other laminectomy procedures at various levels made up 43.5% (n = 20) of surgeries.

Radiographic outcomes

**Figure 2:**
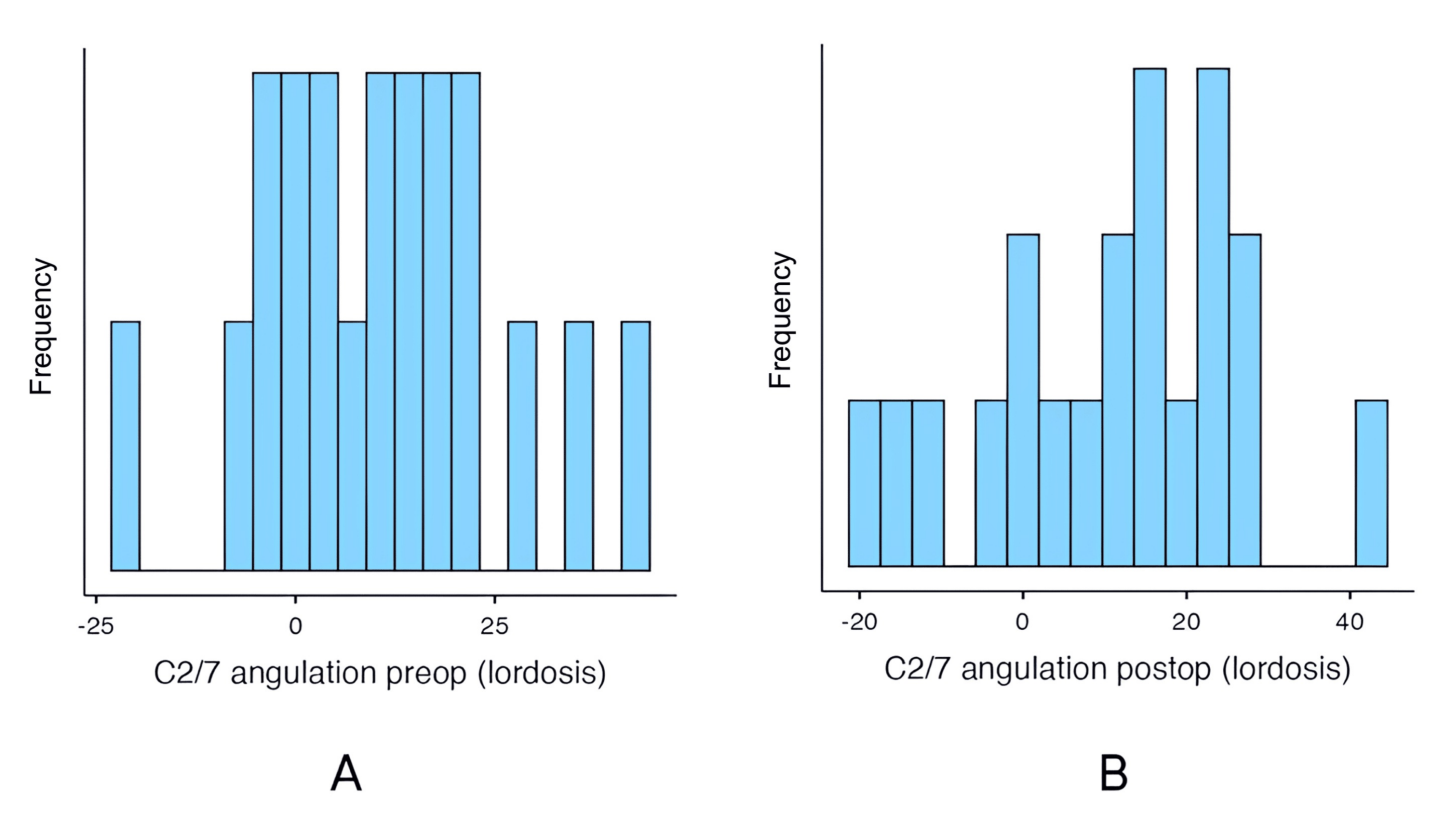
Comparison of preoperative (A) and postoperative (B) C2/7 cervical lordosis angulation in patients undergoing laminectomy for cervical canal stenosis.

One of the primary radiographic outcomes of interest in this study was the progression of kyphosis following laminectomy (Figure 2). Preoperatively, the mean C2/7 angulation (lordosis) was 11.10° (SD = 15.52°, range = -20° to 44°). Postoperatively, the mean C2/7 angulation was slightly reduced to 10.95° (SD = 16.02°, range = -18° to 44°). Linear regression analysis showed a strong correlation between preoperative and postoperative cervical lordosis (R² = 0.733, p < 0.001), indicating that patients with greater preoperative lordosis were more likely to maintain lordosis postoperatively, while those with preoperative kyphosis were more likely to experience further kyphosis.

Follow-up duration and number of levels decompressed were also tested for their interaction with postoperative angulation. Neither of these factors significantly affected postoperative C2/7 angulation (p > 0.05), hence, the number of levels decompressed and follow-up duration were not important predictors of cervical alignment after surgery.

Kyphosis progression

Kyphosis progression was defined as a postoperative reduction in cervical lordosis or reversal into kyphotic alignment (Table 4). In this cohort, kyphosis progression was observed in 40% of patients (n = 8), while 60% (n = 12) maintained or improved their cervical alignment. Despite the radiographic evidence of kyphosis progression, none of the patients reported new or worsening clinical symptoms, such as increased neck pain, radiculopathy, or myelopathy, directly attributable to kyphosis.

**Table 4:** Frequencies of progression of kyphosis.

Progression of kyphosis	Counts	% of total	Cumulative %
No	12	60.0%	60.0%
Yes	8	40.0%	100.0%

This finding indicates that clinical deterioration may not always be accompanied by radiographic progression of kyphosis, especially in older patients with less dynamic cervical spines. The authors then assessed the relationship between the number of levels decompressed and the progression of kyphosis using the chi-square test. In the χ² analysis, the results are insignificant: χ² = 4.86 and p = 0.182. In the simple sense, the number of decompressed levels did not make a clear difference to the likelihood of postoperative kyphosis progression. Additionally, logistic regression models did not reveal any significant predictors of kyphosis progression, including preoperative or postoperative cervical angulation (p > 0.05).

Clinical outcomes

The results of clinical outcomes were measured using the mJOA score, which quantifies the motor, sensory, and bladder function of a patient. The NDI describes the degree of disability related to neck pain. Although the detailed scores of each mJOA and NDI are not mentioned, it has been shown that most patients had clinical improvement after surgery. Lack of postoperative neurological deterioration or significant complications shows that laminectomy alone may be sufficient to restore neurological function in patients with cervical stenosis.

Postoperative complications

In this series, complications after laminectomy were few. There were no cases of postoperative C5 palsy, a complication well known to occur after posterior cervical decompression. No significant postoperative wound infections occurred, and during the follow-up period, none needed to be reoperated for instability or hardware failure issues because no fusion was carried out. This would suggest that laminectomy by itself, without the addition of fusion or laminoplasty, may be able to provide adequate decompression with a low complication rate in carefully selected patients.

Follow-up duration

**Figure 3:**
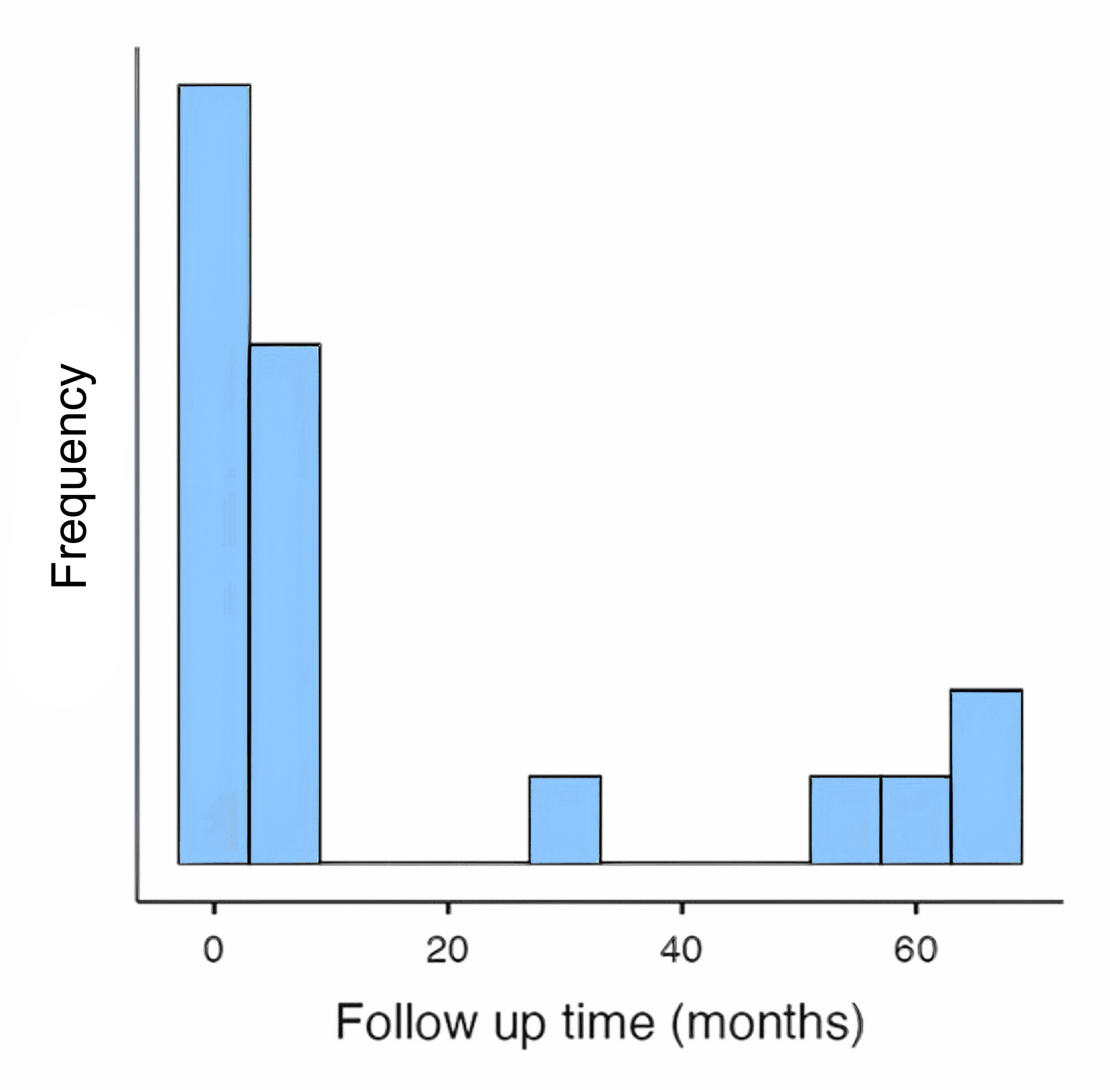
Follow up time in months.

The follow-up duration varied considerably across patients, with a mean follow-up of 16.95 months (SD = 24.17 months, range = 1-67 months) (Figure 3 and Table 5). Multinomial logistic regression revealed that younger patients were more likely to have longer follow-up periods (p < 0.001), suggesting that older patients may not be as likely to return for extended follow-up or may face other health-related challenges that limit long-term follow-up.

**Table 5:** Descriptive statistics of patient demographics, surgical levels, and kyphosis progression outcomes.

Parameter	N	Mean	Median	SD	Minimum	Maximum	W	p
Age	20	62.30	62.00	5.787	50	73	0.987	0.991
No. of levels	20	16.95	4.50	24.171	1	67	0.635	0.013
Follow-up time (months)	20	3.15	3.00	0.875	2	5	0.873	0.013
Name of levels								
C2/7 angulation preoperative (lordosis)	20	11.10	10.50	15.519	-20	44	0.983	0.969
C2/7 angulation postoperative (lordosis)	20	10.95	12.00	16.025	-18	44	0.970	0.750

Statistical analysis

Several statistical analyses were performed to explore the relationships between various factors and clinical outcomes. Chi-square tests were used to examine the relationship between age and the number of decompressed levels, which was not significant (χ² = 47.6, p = 0.255). Similarly, no significant association was found between the number of decompressed levels and postoperative kyphosis progression (p = 0.182). Multinomial logistic regression demonstrated a significant relationship between age and follow-up duration, with younger patients being more likely to have longer follow-up periods (p < 0.001). However, there were no significant associations between age and the number of levels decompressed (p = 0.081). Linear regression models further indicated that preoperative C2/7 angulation was the strongest predictor of postoperative alignment, while other factors, such as follow-up time and number of levels decompressed, did not significantly influence postoperative outcomes.

Synthesis of the key findings

This study supported laminectomy alone as a good surgical treatment for cervical canal stenosis and also showed that most patients had improvement in neurological conditions without any significant complications. While radiographic evidence of the progression of kyphosis was seen in 40% of the cases, none had symptoms related to such a progression, raising the presumption that postoperative kyphosis does not always lead to functional impairment. Indeed, preoperative cervical alignment was found to be the strongest predictor of postoperative outcomes, while other factors such as the number of levels decompressed and follow-up duration did not bear much influence on postoperative alignment and the progression of kyphosis. The absence of major complications like C5 palsy or wound infection gives credence to the safety of laminectomy in selected cases.

## Discussion

The prevalence of degenerative cervical spine disease has been a long-standing issue, as demonstrated by paleopathological studies of skeletal remains from the early Middle Ages. Weber et al. (2003) [9] examined the cervical spines of 196 skeletons from the 6th to 8th centuries AD, finding degenerative changes similar to those observed today, particularly in the C5/6 and C6/7 segments. Interestingly, the study revealed a higher incidence of degenerative disease at the C2/3 facet joints, an area that is not as commonly affected in modern populations. These findings suggest that, despite differences in daily activities and life expectancy, the natural history of cervical spine degeneration has remained consistent over the past 1.5 millennia, indicating the enduring mechanical stress endured by the cervical spine over time.

The purpose of this study was to evaluate the effectiveness of laminectomy alone for cervical canal stenosis in CSM patients. It showed that laminectomy, without fusion or laminoplasty, can provide good neurological results with a very minimal rate of complications in selected cases. Thus, these results support other similar studies done in other countries, supporting the efficiency and safety of decompression by laminectomy, even for resource-constraint healthcare settings like Iraq.

The majority of patients in this cohort experienced significant neurological improvement following laminectomy, as evidenced by improvements in the mJOA score and the NDI. Importantly, there were no reports of postoperative neurological deterioration, further supporting the efficacy of laminectomy as a standalone procedure in this population. These findings are consistent with previous studies that emphasize the benefits of decompressive surgery in halting disease progression in cervical stenosis cases. As revised by de Dios et al. (2022) [10], the national cohort of patients with degenerative cervical myelopathy was studied, focusing on the fact that instrumented fusion at five years was not associated with superior long-term outcomes as compared to laminectomy alone. The absence of significant differences in outcomes at five years further supports indications of laminectomy alone for carefully selected patients, particularly by avoiding complications and increased costs associated with instrumented fusion.

One of the main concerns with laminectomy alone is the potential for postoperative kyphosis. In this study, the mean preoperative C2/7 angulation (lordosis) was 11.10°, which decreased slightly to 10.95° postoperatively. Although kyphosis progression was observed radiographically in a subset of patients, none of these patients reported clinical symptoms related to the radiographic changes. This finding is significant, as it suggests that radiographic progression of kyphosis may not always be clinically relevant, especially in older patients with less dynamic cervical spines. This agrees with the findings of Vedantam et al. (2023) [11], wherein the latter motion preservation surgeries such as laminectomy and laminoplasty resulted in increased stress on the spinal cord, yet their clinical outcomes did not differ significantly from those cases involving procedures for fusion. In a comparison meta-analysis of multilevel cervical myelopathy between laminoplasty (LP) and laminectomy with fusion (LF), Wang et al. (2022) [12] realized that though LP and LF have similar neurological recovery rates, LP exhibited fewer complications like C5 palsy, thus suggesting motion-preserving surgeries may offer clinical advantages in reducing a few postoperative risks. This indeed strengthens the argument that simpler procedures, like laminectomy in isolation, can also yield satisfactory results in many patients, especially when there is no pre-existing instability.

A recent study by Kotter et al. (2020) [13] further strengthens this perspective. Their post-hoc analysis comparing laminectomy with fusion versus laminectomy alone for degenerative cervical myelopathy revealed that while patients treated with laminectomy with fusion demonstrated better functional improvements in the long term, laminectomy alone still provided satisfactory outcomes for certain patients. Specifically, the study highlighted the importance of preoperative alignment and patient-specific factors in determining the need for fusion. Patients undergoing fusion had a longer operative duration but achieved higher scores in functional improvement (e.g., mJOA and Nurick scores) compared to those treated with laminectomy alone. However, these benefits must be balanced against the increased complexity and cost of fusion, especially in resource-constrained settings like Iraq.

The commonest complements to or alternatives for laminectomy alone include laminectomy combined with fusion and laminoplasty. While these techniques nominally reduce the risk of postoperative kyphosis, each has its own complications that include longer operating time, more blood loss, and possible hardware complications. Whereas in laminectomy with fusion, the stress on the spinal cord was reduced, motion-preserving procedures like laminoplasty result in increased off-axis motion with higher stress on the spinal cord during various neck movements such as flexion and lateral bending, a fact discussed by Vedantam et al. in 2023 [11].

Regardless of the biomechanical differences, laminectomy combined with fusion and a standalone laminectomy have been put together, resulting in comparable neurological outcomes in a recent comprehensive review by Paracino et al. (2021) [14]. The review also stated that when fusion diminishes the risks of kyphosis and spinal instability, long-term superior outcomes over the standalone laminectomy were not consistently offered to patients with cervical spondylotic myelopathy. Moreover, the complication rates were significantly higher for fusion than in those treated with laminectomy alone, including axial pain and C5 palsy. In light of these findings, such a result reinforces the concept that simpler or motion-preserving procedures like laminoplasty may be preferred in well-selected cohorts, especially when cervical motion is to be preserved.

Furthermore, Kotter et al. (2020) [13] drew a comparison in functional outcomes between patients undergoing laminectomy with fusion versus those receiving laminectomy alone. While patients undergoing fusion achieved more clinically significant improvements in their mJOA and Nurick scores, simultaneously, the same patients had longer surgeries and an increased need for postoperative management. A key takeaway was that laminectomy alone can still be considered for the patient who does not present with cervical instability, particularly if a simpler procedure with fewer risks is preferred.

Postoperative kyphotic deformities are a significant complication following cervical laminectomy, primarily due to the disruption of the posterior ligamentous complex (PLC). The PLC provides crucial stability to the spine, and its impairment during multilevel laminectomies increases the risk of kyphosis, especially in cases involving extensive resections. Mittal et al. (2024) [15] highlighted that patients undergoing multilevel laminectomies for conditions such as intradural tumors frequently develop cervical kyphosis, leading to progressive neurological decline and requiring further interventions like posterior cervical fusion to correct the deformity.

Moreover, one more recent study by Pettersson et al. (2023) [16] outlined that cervical kyphotic deformity represents a frequent complication, while kyphotic deformity in up to 21% of all patients may be observed after laminoplasty. Both studies underlined the importance of preoperative planning, supported by the need for posterior fixation techniques with the aim of preventing post-laminectomy kyphosis and maintaining spinal stability.

The limitations of this study need to be realized. First, the present study is a retrospective cohort study in a single center and thus probably cannot provide findings that can be generalized in the population or outside a healthcare setting than those in the area of the study. Moreover, with the limited sample size of 46 patients participating, some analysis targets tended to have low statistical power, such as rare complications and slight differences in outcomes. Apart from the fact mentioned above, the follow-up period is different; this might decrease the possibility of detecting the investigation about long-term complications, such as kyphosis progression, which may show up over time.

This therefore supports the assertion that laminectomy alone is a good and viable surgical option in patients with cervical canal stenosis, especially in resource-poor settings. Minor kyphosis progression was realized; however, this did not lead to clinical deterioration, which again suggested that laminectomy without fusion may be adequate in many patients. These findings are consistent with a number of international studies and emphasize the key role of patient selection in optimizing surgical outcomes. A randomized controlled trial and further comparative studies among available surgical techniques will help to further hone the indications for laminectomy alone and consequently further improve the outcomes of the patients.

## Conclusions

This study justifies that laminectomy alone is a considerably effective and safe option in cervical canal stenosis due to spondylotic changes, particularly in resource-limited settings. Many patients benefited with significant neurological improvement; however, the progression of kyphosis occurred in some, which did not clinically manifest symptoms. Concerning the postoperative outcome, preoperative cervical alignment was an important determinant factor, reflecting the importance of adequate case selection. Yet, laminectomy alone, without fusion or laminoplasty, is indeed a reasonable alternative for adequately selected patients in a cost-effective manner. Larger, multicenter trials with longer follow-up periods are needed to confirm these results and enhance the generalizability. Randomized controlled trials will compare laminectomy alone with other techniques, such as laminoplasty or fusion, to further elucidate the best surgical strategy. The inclusion of patient-reported outcomes and further analysis of preoperative alignment may further refine patient selection and optimize surgical outcomes.
